# Engineering of fluorescent or photoactive Trojan probes for detection and eradication of β-Amyloids

**DOI:** 10.1080/10717544.2020.1785048

**Published:** 2020-06-29

**Authors:** Amal A. Aziz, Rafat A. Siddiqui, Zareen Amtul

**Affiliations:** a Sir Wilfrid Laurier Secondary School, Thames Valley District School Board, London, Canada; b Nutrition Science and Food Chemistry Laboratory, Agricultural Research Station, Virginia State University, Petersburg, VA, USA; c Department of Chemistry and Biochemistry, University of Windsor, Windsor, Canada

**Keywords:** Alzheimer’s disease, Trojan horse, β − amyloid, blood-brain barrier, photosensitizer, quantum dots, smart drugs

## Abstract

Trojan horse technology institutes a potentially promising strategy to bring together a diagnostic or cell-based drug design and a delivery platform. It provides the opportunity to re-engineer a novel multimodal, neurovascular detection probe, or medicine to fuse with blood-brain barrier (BBB) molecular Trojan horse. In Alzheimer’s disease (AD) this could allow the targeted delivery of detection or therapeutic probes across the BBB to the sites of plaques and tangles development to image or decrease amyloid load, enhance perivascular Aβ clearance, and improve cerebral blood flow, owing principally to the significantly improved cerebral permeation. A Trojan horse can also be equipped with photosensitizers, nanoparticles, quantum dots, or fluorescent molecules to function as multiple targeting theranostic compounds that could be activated following changes in disease-specific processes of the diseased tissue such as pH and protease activity, or exogenous stimuli such as, light. This concept review theorizes the use of receptor-mediated transport-based platforms to transform such novel ideas to engineer systemic and smart Trojan detection or therapeutic probes to advance the neurodegenerative field.

## Introduction

The defective blood-brain barrier (BBB) function (Amtul et al., 2018) or vascular phenotype or both leads to dysregulated cerebral blood flow (CBF) (Yang et al., 2014) resulting in proteinaceous storage disorders in the brain and cerebral vessels (Amtul et al., 2019; 2020) of Alzheimer’s patients (Zlokovic, 2008). Aggregation of monomeric amyloid β-peptide (Aβ) into Aβ fibrils/oligomeric species of Aβ plaques is a critical step in the pathophysiology of Alzheimer’s disease (AD) (Meinhardt et al., 2009). Single protein or gene delivery to combat a single drug target in multifactorial Alzheimer's disease (AD) has thus far not been very successful, resulting in the advent of co-expression of multiple-target or multi-function therapeutic proteins or genes (Aziz & Amtul, 2019). However, the main hurdle current AD diagnostics and therapeutics face is the impaired penetration of neuroactive probes through the BBB. This problem is also partly the reason for the failure of the majority of AD trials (Amtul et al., 2011; Cummings et al., 2014; Amtul, 2016; Amtul et al., 2018).

An unconventional approach could be to ferry these multiple-target diagnostics or therapeutics via the receptor-mediated transcytosis across the BBB by fuzing them with BBB molecular Trojan horse (Pardridge, 1986). The molecular Trojan horse is a term used for the endogenously synthesized monoclonal antibodies (MAb) to the specific receptors found on the endothelial cells of the BBB (Pardridge, 1986), such as transferrin receptors (TfR) (Mura & Couvreur, 2012). These receptors are an essential component of the receptor-mediated transport system on BBB. Most importantly, AD neuropathology doesn’t impair the TfR-mediated uptake at the BBB (Bourassa et al., 2019). The TfRMAbs act like shuttle peptides or lower-affinity ligands for their receptors to transport cargo from blood to the brain. Many attempts have been made to manipulate this natural phenomenon by attaching a detection or therapeutic probe to these Trojan horses for their safe entry to the brain (Pardridge, 1986). It is similar to the trick used by the Greek soldiers to enter the city of Troy in Trojan War by hiding a select force of soldiers inside a huge wooden Trojan horse, which was pulled by the Trojans into the city as a winning plaque, the hidden soldiers, later on, destroyed the city of Troy (Sparkes, 1971).

In this regard we have already reported that an ideal Trojan drug should consist of a chimeric bispecific (BsAb) MAb (against β-amyloid (Aβ) and TfR), conjugated to a fusion drug that should have at least three components: (i) an Aβ disaggregating component, (ii) an Aβ clearing component to eliminate the disaggregated Aβ from the brain to the blood, and (iii) a vascular component for healing the vascular endothelium (Aziz & Amtul, 2019). Further to what we have reported already (Aziz & Amtul, 2019), the primary purpose of the present concept review article is to additionally draw some light on the type, design, functioning, more specifically on the engineering of some of the novel fusion molecules, from the plethora of available molecules that could be attached to a Trojan horse for either detection or therapeutic purposes.

For instance, Trojan probes could be designed to carry a smart prodrug or detection probe that could be switched into an active form by an alteration in the diseased tissue environment, such as lower pH (Wang et al., 2015), redox state, enzyme activation/conformation (Fu et al., 2014), elevated temperature or magnetic field (Silva et al., 2013). Similarly, photosensitizers: that are capable of producing reactive oxygen species, such as cytotoxic singlet oxygen (^1^O_2_) after absorbing external light, could also be fused to the Trojan horse. Singlet oxygen then could effectively disintegrate the Aβ plaques locally. Here, we also proposed a mechanism to eliminate the dependency of these photosensitizers on an external light source; instead, they could be designed to carry their own light source. Such activatable, switchable, smart Trojan probes could assure the safe passage to the site of injury without causing any harm to the healthy tissues/cells on their way. Besides, such probes offer an external spatial and temporal control over their activities. Moreover, as an additional feature, the use of CdSe/ZnS quantum dots (QDs) (Mura & Couvreur, 2012) could enable a Trojan therapeutic or detection probe to do dynamic *in vivo* monitoring of anti-Aβ-Aβ plaques complexes or therapies via functional near-infrared region spectroscopy.

We firmly believe that the mechanistic studies in the aforementioned Trojan horse designs are predestined, and awaiting the attention of the researchers to be delineated to bring revolution in this drug delivery field of neurodegenerative disorders.

## Central skeleton of a Trojan probe

**Figure 1.**
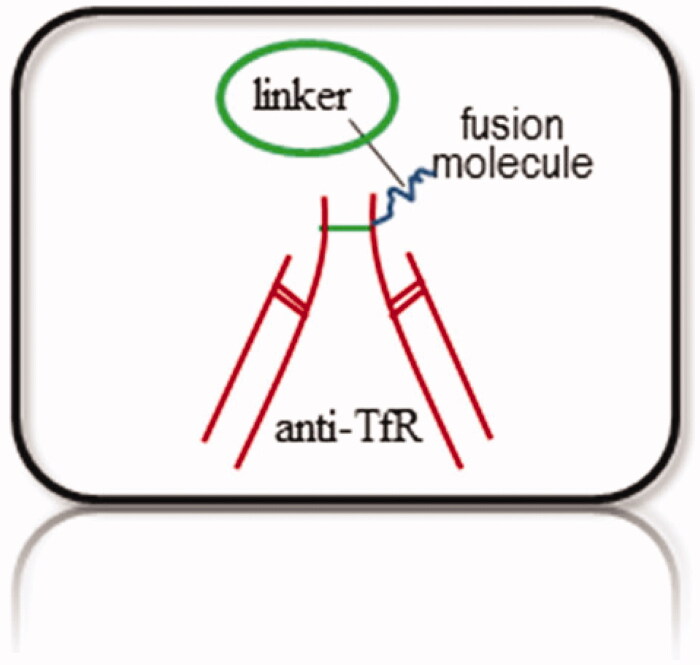
Trojan Probe. The central skeleton of a Trojan probe should consist of at least three components; a TfR monoclonal antibody (TfRMAb), a high yield coupling and cleavable linker that attaches TfRMAb to the fusion protein, and finally the fusion protein that is unable to cross BBB at its own. A bi-functional Trojan horse:anti-Aβ chimera could be synthesized by fusing the one arm of TfRMAb to one arm of anti-Aβ to make a monovalent chimeric bispecific antibody that simultaneously recognizes two different targets with its two arms (Atwal et al., 2011; Yu et al., 2011).

Trojan horse technology is a highly multidisciplinary field that could involve chemists, physicists, biologists, engineers, and physicians who are continuously pursuing to propose, produce, isolate and characterize new candidate molecules to treat brain disorders, including AD. The central skeleton of such Trojan probes should consist of at least three components; (i) TfRMAb with known pharmacokinetics, (ii) a high yield coupling and cleavable linker that attaches TfRMAb to the drug and finally (iii) the fusion protein (for detection or treatment) that is unable to cross BBB at its own (Figure 1).

### Transferrin receptor monoclonal antibody

To make a BBB crossing probe it is essential to synthesize a recombinant molecular Trojan horse as described in detail (Pardridge, 2008). OX26 antibody is the most comprehensively investigated TfRMAb (Lee et al., 2000), especially in studies with rats. OX26 recognizes an epitope on the extracellular site of the rat TfR, distinct from its natural ligand binding-site for transferrin peptide (Tf), thus avoiding the binding competition between the fusion protein and Tf for the Tf binding-site (Lee et al., 2000). Given the fact that the OX26 antibody cannot cross-react with the mouse TfR, the 8D3 antibody is recommended to be used as a mouse BBB targeting Trojan horse (Lee et al., 2000). Likewise, the RI7-217 antibody has also shown high transport across the mouse BBB (Johnsen et al., 2019).

### Linker

Various approaches could be used to attach the drug (such as cloned fusion proteins) to the Trojan horse either via genetic engineering or by employing chemical linkers. These chemical linkers could be of any type, such as non-cleavable strong bonding (amide or biotin) linkers, cleavable stimuli-responsive temporary bonding (disulfide or hydrophobic) linkers or PEGylated linkers (Couvreur, 2013). These linkers ensure the safe intracellular delivery of fused proteins to the target after separation from the Trojan horse, as well as keep their biological activity intact. Further, an azide moiety could be anchored at the terminal of such molecules for coupling to a fluorescent probe (such as Alexa Fluor 555) or an alkyne functionality via click-chemistry reactions or quantum dots (QDs) to detect or image the Trojan horse and fusion protein cleavage by illuminating fluorescence (Prescher & Bertozzi, 2005; Zhuang et al., 2013). The type and length of the linker molecule are of critical importance (D’Angelo et al., 2013), as discussed below:

#### Disulfide linker

**Figure 2.**
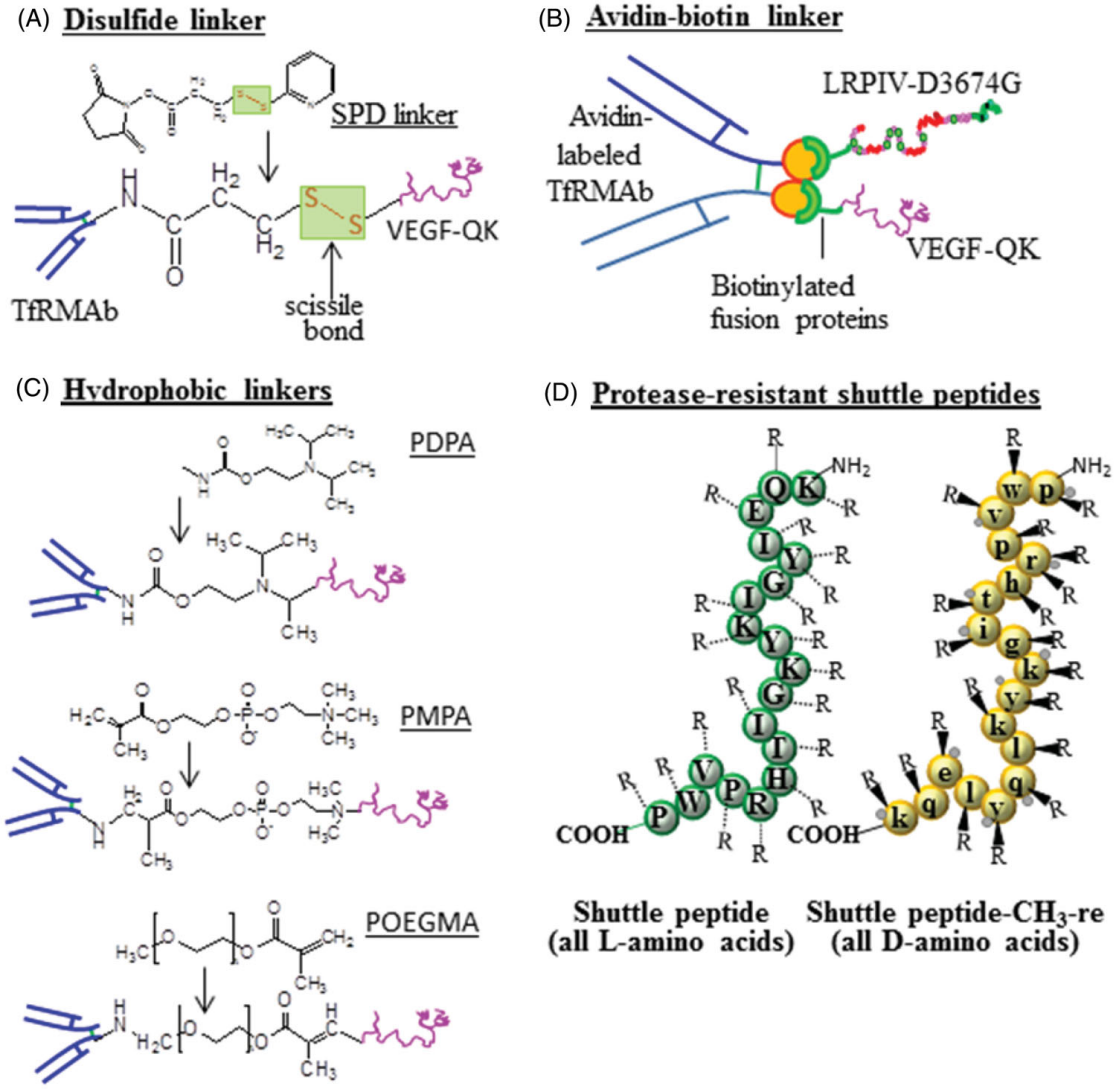
Linkers and protease-resistant Trojan horse or shuttle peptides. TfRMAb is activated by incubation with succinimidyl 3-(2-pyridyldithio) propionate (SPDP), conjugates are isolated and incubated with the purified fusion protein (VEGF-QK). A scissile disulfide linkage bonds the activated TfRMAb and the fusion protein (A). Poly (2-(diisopropylamino) ethyl methacrylate) (PDPA), peroxomono-phosphoric acid (PMPA) and hydrophilic linker poly[oligo(ethylene glycol) methyl methacrylate] (POEGMA) are used in the same way (B). Schematic shows the avidin-biotin bonding between the monobiotinylated fusion proteins (LRPIV-D^3674^G, VEGF-QK) and the avidin-labeled TfRMAb (C). Blood circulation time or protease-resistance of shuttle peptides can be enhanced by their surface functionalization by methylation or by *retro-enantio* (re) sequence or altered stereochemistry of all L-amino acids to all D-amino acids in reverse sequence order but preserved topochemical features (D).

To make an amine-to-sulfhydryl conjugated scissile disulfide crosslinker, TfRMAb could be activated with succinimidyl 3-(2-pyridyldithio) propionate (SPDP) in a controlled reaction (Chen et al., 2013). Activated TfRMAb is then reacted with the unconjugated cysteine residue near the *N*-terminus of the fusion protein via the cleavable disulfide bond (Barat et al., 2009; Feng et al., 2013). TfRMAb-fusion protein complex then binds the TfR on BBB, while there should be no uptake of the unbound fusion protein in a pharmacologically effective concentration. Once the Trojan horse-fusion protein complex is internalized and trafficked to sorting endosomes, reducing the environment of the endosomal compartment should trigger the cleavage of the self-scissile disulfide bond to allow the separation of TfRMAb-fusion protein complex (Chen et al., 2013). Fusion protein then exits the endosomal compartment, enters the nucleus, etc. (Figure 2(A)).

#### Hydrophobic linker

Similarly, a pH-sensitive hydrophobic PDPA; poly(2-(diisopropylamino)ethyl methacrylate) linker molecule (pKa 6.4) (Mu et al., 2019) could also be used to temporarily bond a fusion protein and TfRMAb. A decrease in the local (endosomal) pH below the pKa of PDPA renders the tertiary NH_2_ moiety and chain of the PDPA vastly protonated, and hydrophilic, respectively, in their weak cationic polyelectrolyte forms (Mu et al., 2019). As a result, TfRMAb-fusion protein complex cleaves to produce TfRMAb and fusion protein. This, in turn, initiates an increase in the endosomal osmotic pressure to briefly lyse its membrane and releasing its content into the cytosol. This strategy has already been used to enable the cytosolic delivery of several cargos and molecular species, including anticancer drugs (Colley et al., 2014), antibiotics (Wayakanon et al., 2013), nucleic acids (Wang et al., 2012), and proteins (Wang et al., 2012; Canton et al., 2013).

Similarly, PMPC; poly[2-(methacryloyloxy)ethyl phosphorylcholine], which constitutes only pH-labile functional moieties, has demonstrated sensitivity to both pH and temperature (Mu et al., 2019). Also, a matrix metalloproteinase2/matrix metalloproteinase9 (MMP2/MMP9) degradable and thermo-responsive hydrophilic POEGMA; poly[oligo(ethylene glycol) methyl methacrylate] have been synthesized by controlled polymerization technique atom transfer radical polymerization and researched for possible drug delivery applications (Jung et al., 2007) (Figure 2(B)).

#### Avidin-biotin linker

IgG, as a fusion protein, cannot be engineered for certain biopharmaceuticals, such as oligopeptides, sequence-specific short interfering RNA, or antisense drugs (Pardridge, 2008). The *in vivo* delivery of such biotherapeutics or diagnostics through the BBB could be made possible by highly stable avidin-biotin bonding between the monobiotinylated fusion protein (biotin moieties are attached using –SS- or –XX- linkers) and the (streptavidin or neutral light) avidin-labeled TfRMAb (McMahon, 2008). Where SS may be a cleavable disulfide (thioether) 8-atom spacer and XX is a non-cleavable (amide) 14-atom spacer linker (McMahon, 2008). Due to a very strong (kD 10–15 M) affinity and dissociation (half-life 89-day) (Green, 1975) of avidin-biotin complex, the biotinylated fusion protein is instantaneously captured by the avidin-labeled TfRMAb (Boado et al., 2008). Placement of a biotin molecule at the 3′ end of the phosphodiester antisense oligodeoxynucleotides not only facilitates the attachment but also renders its degradation by the 3′-exonuclease (Asseline et al., 1984). Nearly any mono-biotinylated fusion protein or biopharmaceutical could be carried to the brain with the avidin-labeled TfRMAb that could serve as a universal Trojan horse. Additionally, avidin is shown to elicit no immunologic reactions when administered to humans (Samuel et al., 1996) (Figure 2(C)).

### Fusion molecule

Depending on the type of fusion molecule, a Trojan probe could be designed to detect and/or disaggregate the β-amyloids in an AD brain. A fusion molecule could range from a protein to an organic compound. There is almost an endless number of BBB-restricted neuropharmaceuticals (including recombinant MAbs, proteins, lysosomal enzymes, and RNAi or antisense molecules) that could be re-engineered for fusing to the genetically engineered molecular Trojan horses. New chemical entities could also be created that will allow cerebral drug delivery to short circuit cerebral drug discovery programs for the diverse nature of brain disorders. In the case of proteins, several multiple protein/genetic engineering methodologies can be employed to develop TfRMAb-fusion protein complexes to let through the BBB.

#### Synthesis of fusion molecules

Since the gene therapy field directed to treat brain ailments remain perplexing owing to the limited access of gene-vectors via BBB into the brain. Therefore, in addition to using the Trojan horse to deliver protein payloads, exogenous genes of such proteins could be delivered by synthesizing via molecular cloning using plasmid DNA encoding the genes. However, co-transfecting two or more different constructs might unintentionally lead to uneven and inconsistent product-delivery into the cells owing to the interfering promoters with varying strength. To rule this out, open reading frames expressing multiple proteins in equimolar amounts should only be controlled by one promoter. There are reports to detect modifiable epitopes on adeno-associated virus type 2 vectors via *in vivo* phage panning to assist in endothelium-specific delivery of genes (Geoghegan et al., 2014). Briefly, the coding sequences of a candidate drug or detection probe-gene could be amplified by polymerase chain reaction under the ubiquitous promoter cytomegalovirus (Gray et al., 2011) or brain-specific glial fibrillary acidic protein (GFAP) promoter at the 5′ end with specific primers from a mouse cDNA library, separated by 16-amino-acid residue Gly-Ser-Gly p2a linker sequence (A T N F S L L K Q A G D V E E N P G P) (Kuzmich et al., 2013). The mutations could be inserted in the fusion proteins by using site-directed mutagenesis and cloned into the mammalian expression vector/plasmid, such as pAcGFP1-N1 (Ahsan & Gore, 2011). The primers should contain specific restriction sites that are not present in the coding sequence and are unique sites of the pAcGFP1-N1 vector. The reverse primers must not contain the stop codon.

Beside molecular cloning, combinatorial chemistry (Vendrell et al., 2012), virtual screening (Ma et al., 2013), pharmacophore modeling (Yang, 2010), fragment-based approaches (Scott et al., 2012), and network-based approaches (Harrold et al., 2013), could also be used to synthesize the TfRMAb conjugated fusion proteins. A few of these approaches have been briefly outlined below.

## Engineering of fluorescent Trojan detection probes

The Trojan probes could also be designed to diagnose AD in the earlier stages by focusing on specific vascular or metabolic factors or Aβ species (such as Aβ oligomers) that could prove to be an attractive biomarker for diagnostic purposes.

### Oligomer-specific fluorescent chemical probe

Fluorescent chemical probes that preferably identify oligomeric assemblies over monomeric or fibrillar aggregates of Aβ peptides could be attached to a Trojan horse as a fusion molecule. By conjugation with Trojan horse, fluorescent chemical probe, generated in-house through combinatorial chemistry (Vendrell et al., 2012), not only could cross BBB but also detect Aβ oligomers *in vivo*. Beside virtual screening (Ma et al., 2013); pharmacophore modeling (Yang, 2010); fragment-based approaches (Scott et al., 2012); and network-based approaches (Harrold et al., 2013); oligomer-specific fluorescent chemical probes could also be synthesized via diversity-oriented fluorescent library approach (DOFLA) (Yun et al., 2014) as well as imaging-based, high-content screening or biology-oriented synthesis (BIOS) (Wetzel et al., 2011). DOFLA and BIOS use the structural properties (i.e. chelating features, electrostatic charges, environmental-sensitivity) of the potential targets to modify or derivatize the side chains of different fluorescent dye backbones or fluorogenic scaffolds without affecting the photophysical properties of the fluorophores to provide new chemical pools of potential sensors for detection as well as treatment purposes (Wetzel et al., 2011; Yun et al., 2014).

Additionally, instead of synthesizing small molecules in the labs and do real-time screening to examine if the fluorescent chemical probe could save neurons, in-house, *in silico* virtual computational modeling techniques (among others) could also be used (Aziz & Amtul, 2019). It offers a very effective alternative to predict, based on a fluorophore’s modified size and shape, whether it will bind the target or cross the barrier. These probes natively exhibit optical properties that are suitable for noninvasive imaging of AD pathological changes. Such fluorescent chemical probes also demonstrate a dynamic ability to monitor oligomers during *in vitro* fibrillogenesis of Aβ peptides (Teoh et al., 2015), as Aβ is induced over time to form oligomers and eventually fibrils. Furthermore, delivering an activatable chemically reactive group that recognizes and makes an irreversible covalent linkage with a binding sequence on Aβ could simply switch a low-affinity ligand into a high-affinity prodrug.

### Oligomer-specific fluorescent peptide probe

Besides, large antibodies and chemical probes, peptide-based synthetic fluorescent probes (including ligands for BBB receptors) could also be developed based on the unique structural properties or functions of Aβ (just like Aβ-oligomer targeting probes) using DOFLA or combinatorial chemistry approach (Teoh et al., 2015). However, smaller peptide probes offer numerous advantages over antibodies. They could be synthesized and modified easily, and have a lower tendency to invoke an immunogenic reaction, especially those without a rigid structure (Uhlig et al., 2014). Besides, smaller peptides result in a higher target-to-background ratio due to their rapid blood clearance. For instance, PG44, an engineered Aβ construct, created by integrating the conformation labile; FlAsH with the Aβ oligomer self-recognizing sequences can rapidly detect Aβ oligomers specifically and quantitatively (Hu et al., 2012). Even a conformation-switching probe (PG65) that detects oligomers formed by the wild-type α-synuclein has been designed for engineering the structural flexibility of an intrinsically disordered protein (IDP) variant to α-synuclein conformation-sensitive biarsenical fluorescent dye (Hernandez et al., 2013). The advantages of such peptides are two-fold: they are low cost due to the small size, and second, they are highly specific (Uhlig et al., 2014). After anchoring with Trojan horse, these peptides with medium to low affinities for BBB receptors (Uhlig et al., 2014), are also capable of crossing the BBB and be observable by intracranial two-photon microscopy.

The fact that peptides are amenable to synthesize chemically allows the introduction of a varied range of unnatural modifications and functional moieties for site-specific conjugations to nano-carriers. For instance, peptides with binding affinity for Aβ loop-forming residues; referred to as the Aβ linkers or blockers that are especially found in particular β-turn conformations as described above, change Aβ propensity to form oligomers or fibrillar aggregates (Larini & Shea, 2012). Synthesis of such Aβ-linker peptides conjugated with a fluorophore also represents a novel approach to detect as well as control Aβ oligomerization (Hu et al., 2012).

## Engineering of photoexcited Trojan therapeutic probes

At present, photodynamic therapy (PDT) for AD treatment is still in its infancy and is not considered a mature therapeutic method. Nevertheless, photosensitizers, such as meso-tetra (4-sulfonatophenyl) porphyrin (Lee et al., 2015), rose Bengal (Lee et al., 2015), and tri-s-triazine (g-C_3_N_4_) (Chung et al., 2016) have been successfully used to inhibit *in vitro* aggregation of Aβ monomers. Porphysomes (self-assembled nanostructures from porphyrin-lipid building blocks) are also an interesting example of a theranostic PDT agent (Charron et al., 2015) that could be used to inhibit Aβ just like porphyrins. Yet, an Aβ aggregation inhibitor with an outstanding photo-stability, high ^1^O_2_ quantum yield, and biocompatibility, as a new generation of multi-function bio-therapeutics is greatly anticipated.

### Photosensitizers

**Figure 3.**
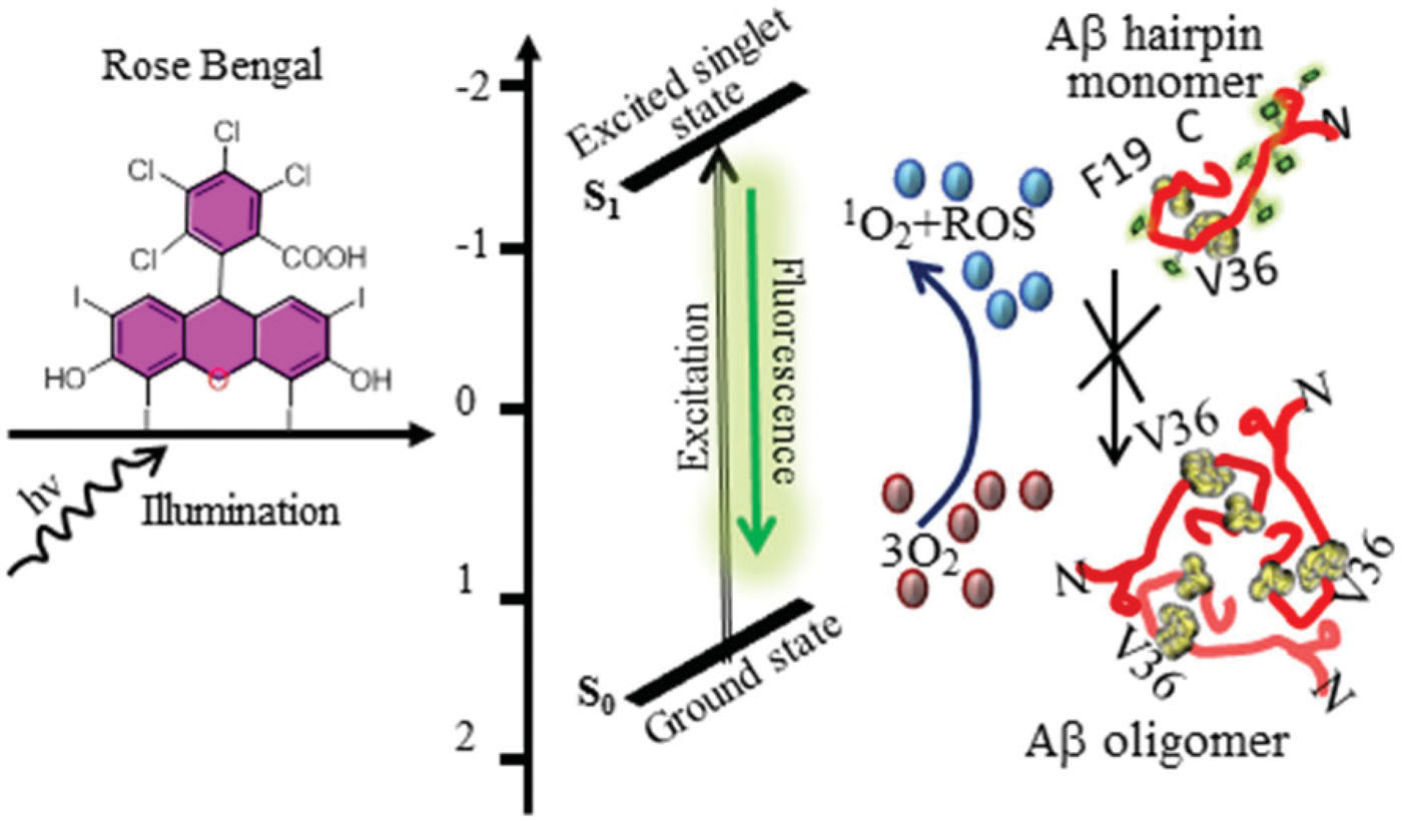
Photosensitizers. Photoinduced or irradiated electrons of the photosensitizer (here rose Bengal) generate excited state ^1^O_2_ and reactive oxygen species (ROS). When excited from the ground (S_0_) to excited (S_1_) state, it results in the photo-oxidation of Aβ peptides blocking their aggregation into the Aβ oligomers. While coming back to the ground state, it also emits fluorescence.

Photosensitizers (nontoxic dyes) are biocompatible organic compounds that, upon illumination, produce reactive oxygen species, such as ^1^O_2_ (Dougherty et al., 1978) under an excitation state by irradiating oxygen gas (photo-oxidation). This feature of photosensitizers could be used to kill (usually cancerous) cells locally, vessels, and tissues (Celli et al., 2010) in PDT, and make them a right candidate for multimodal imaging and image-guided therapy (Figure 3).

While limiting illumination to the specific site (like in tumor) permits a certain level of control over photosensitizer selective activation. Conversely, PDT application is not possible in multifarious anatomical locations, such as the thoracic, abdominal cavities, or nervous tissue. Also, in circumstances where the selectivity and the short radius of action (0.01–0.2 µM in cells) of ^1^O_2_ (Moan & Berg, 1991) and lifetime (∼10–320 ns) is insufficient. Owing to the need for imaging and treatment of deeper tissues to widen the spectrum of PDT applications, targeted delivery of photosensitizers becomes essential. To do this, Trojan horse technology could be integrated with the principle of PDT. Conjugates of photosensitizer-TfRMAb could be prepared via click chemistry. Although the fluorescence could rather quench, the conjugates would have identical ^1^O_2_ quantum yields as to free photosensitizer.

#### Stimulus-triggered probes

While microendoscopic techniques could also be used to deliver light and guide the therapy. Still, due to the limited light penetration into the nervous tissue, stimulus-triggered production of ^1^O_2_ in a non-photochemical reaction (Mor et al., 2014; Koh et al., 2016), in addition to light-triggered, would rather prove to be more effective in AD to achieve higher targeting efficiency and reduce systemic toxicity.

This could be achieved by constructing agents (such as pheophorbide *a* (Mor et al., 2014)) that are activatable by the alterations induced by the amyloid plaque-associated proteases or pH. In such cases, ^1^O_2_ producers could serve as ‘prodrugs’ that are activated into active drugs (like protoporphyrin IX (Lee et al., 2015)) upon stimulus activation of the internal light source (such as luciferase reaction) conjugated to the Trojan drug (Aziz & Amtul, 2019). Such stimuli-sensitive prodrugs promise increased selectivity for plaque-enriched regions while sparing plaque-free areas of the brain.

**Figure 4.**
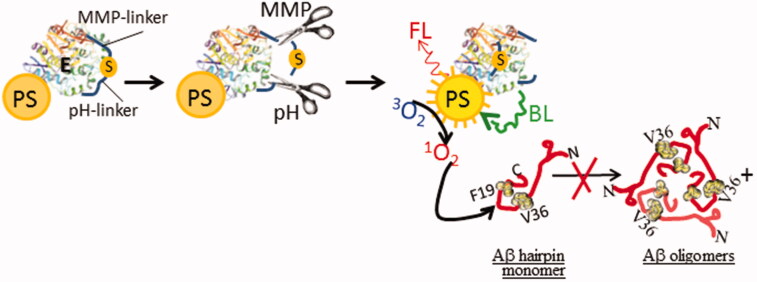
Dual pH- and protease-sensitive probe. The substrate (S) is kept away from the luciferase enzyme (E) by the protease (matrix metalloproteinase; MMP)- and pH-sensitive linkers attached to the enzyme. When the prodrug meets both the conditions, usually in actively multiplying the Aβ brain, the S is liberated from its linkers to bind to the enzyme active site. Bioluminescence (BL), produces as a result of enzyme-substrate reaction, activates the photosensitizer (PS) conjugated to the enzyme to produce ^1^O_2_, which then prevents the polymerization of Aβ monomers into the oligomers.

Such probes should be nontoxic and non-fluorescent (optically silent) in their native state, but stimulus-mediated activation should prompt the prodrug to produce ^1^O_2_ with or without fluorescent (Aziz & Amtul, 2019). This allows diagnostic visualization and therapeutic application by selectively oxidizing the Aβ monomers to disturb their assembly before they are transformed into amyloid oligomers, fibrils, or plaques (Figure 4).

#### pH-sensitive probes

Since the pH of the plaque region is slightly more acidic (∼pH 5.8) than the pH of the healthy regions (∼pH 7.4) (Fraser et al., 1991; Su & Chang, 2001). Thus, an environmentally sensitive TfRMAb-prodrug probe based on pH-responsiveness or acid-catalyzed hydrolysis mechanism could be actively researched as a promising plaque targeting strategy as outlined in Figure 4. For instance, an acid-labile, self-quenched hydrazone linkage (Galande et al., 2006) or probe that also fluorescence in the acidic environment could be attached to the C-terminus of the prodrug sequence. To do this a di-aldehyde peptide is prepared in the presence of a disulfide bond by selective and mild periodate oxidation of the 2-amino alcohol of serine residue (Amore et al., 2013). Two similar fluorochrome hydrazide derivatives are next connected to the di-aldehyde peptide to form hydrazone linkage. This probe shows weak fluorescent at physiological pH but fluoresces 3 times more in an acidic environment (pH 4.5). Such drug could be synthesized in a way that when it encounters negatively charged cell membranes or the acidic environment of plaque containing-brain parenchyma, then it exhibits enhanced cellular internalization into the diseased neurons, to get access to the intracellular Aβ for disaggregating its assembly.

#### Protease-sensitive probes

Enzymatically (proteolytically) activated peptide-based probes could also be developed as potent suppressors of Aβ aggregation to prevent its buildup effectively. This concept exploits the fact that capillary basement membranes and connecting tissues are degraded during the Aβ invasion process (Inoue, 2001). Many proteolytic enzymes are supposed to be, and few of them are, the part of this process, such as MMP endopeptidases that degrade the proteins contained in the extracellular matrix called matrixins (Nagase et al., 2006), and their expression should also possibly increase actively in the milieu of plaque formation. Thus, they are the promising enzymes to be exploited for lead development, modification, delivery, imaging, as well as sensing purposes.

There has not been much research done on distinct proteases found in the milieu of plaque formation and invasion. However, amyloid plaque-associated proteases should supposedly function at multiple stages of plaque progression, affecting plaque seeding, growth, neovascularization, and intravasation. Prior reports have shown the Aβ-induced up-regulated expression of various proteases (e.g. MMPs (Roher et al., 1994; Deb & Gottschall, 1996), neprilysin (NEP) (Mohajeri et al., 2002), endothelin-converting enzyme-2 (ECE-2); that cleaves big endothelin to produce the vasoconstrictor endothelin-1 and thus reduces cerebral blood flow (Palmer et al., 2009), insulin-degrading enzyme (IDE) (Vepsäläinen et al., 2008) in the vicinity of amyloid plaques (mostly to degrade Aβ). These plaque or Aβ-associated proteases could potentially serve as the activators of the proposed probes (Figure 4).

Alternatively, a biodegradable poly-l-lysine grafted with monomethoxy-PEG (PEG; L-PGC) backbone specific for a targeted protease and coupled with multiple fluorescent chlorin e6 (Ce6) molecules is attached to the carboxyl-terminal of a prodrug to synthesize protease activatable probe (Tung, 2004). Typically, a fluorochrome or photo-excited prodrug is conjugated to the carboxyl-terminal of a peptide sequence, which is amenable to a proteolytic attack. A distinct feature of such types of probes is that the un-cleaved peptide substrate should have no effects and emits no fluorescence until the plaques-associated protease releases the substrate by cleaving the bond between the peptide C-terminus and substrate. Fluorescence (due to the released Ce6) or ^1^O_2_ generation is expected to increase on protease-mediated stimulation of the light source and the release of the prodrug. For instance, cathepsin-B (L-SR15) and thrombin-sensitive photosensitizers have been used for increased selectivity in atheromata and thrombin-rich synovial sites of rheumatoid arthritis (Gabriel et al., 2012), respectively.

#### Dual pH- and redox-sensitive probes

The specificity of prodrug activation could be increased significantly by synthesizing a prodrug with dual cross-linked architecture, such as both pH- and redox-sensitivities contained in a central micelle (Wang et al., 2013). In this way, a prodrug can only be activated when stimulation for both pH and reducing conditions is present. In cases, when low pH or redox is applied independently, no disassembly or activation or release of encapsulated hydrophobic prodrug should be noticed as the probe is still cross-linked via disulfide or imine bonds. The effective activation of such a double-sealed prodrug could only be initiated by concomitantly applying low pH and reducing conditions. To do this, a prodrug molecule could initially be bonded to a hydrazine functionalized block copolymer poly(ethylene oxide)*-b-*poly(methacrylic acid) (PEO*-b-*PMAA) via hydrazine formation (Zhuang et al., 2013). Then the prodrug-linked block-copolymer is deposited autonomously into a stable micelle, which is cross-linked more by reacting with the remaining hydrazine functionalities by adding di-thiodiethanoic acid (Talelli et al., 2015). Upon subjecting the cross-linked micelle to neutral pH, no significant release of prodrug should be observed in reducing environment, because the hydrazone linkage is still intact. However, the reducing disulfides uncross-link the micelles. Similarly, the prodrug will not be activated in an acidic environment, despite the cleavage of the polymer prodrug backbone because the prodrug is still trapped within the cross-linked micelle.

#### Graphene quantum dots

In PDT, many researchers have prepared conjugates between QDs and various photosensitizers (Zhou et al., 2015). QDs, in addition to magnetic resonance imaging and positron emission tomography (PET) contrast agents, can also help to visualize the Trojan horse accumulation in deep tissues. In this respect, graphene QDs (GQDs) are very effective in mediating PDT on their own without adding photosensitizer (Ge et al., 2014). It is reported recently that PEG derivatives passivated GQDs are capable of generating ^1^O_2_ upon blue light irradiation (Markovic et al., 2012) via a multistate sensitization process with ∼1.3 quantum yield, nearly twofold as much as of all available state-of-the-art organic PDT agents (Ge et al., 2014). The GQDs are also characterized by a broad absorption band that spans the UV, entire visible, and robust deep-red emission regions peaking at 680 nm (Ge et al., 2014). This simultaneously allows imaging and highly efficient cytotoxic therapy better than a trivial PDT agent in terms of photo-stability, water dispersibility, biocompatibility, and ^1^O_2_ quantum yield. In neurodegenerative PDT research, instead of photosensitizers, stimulus-activated GQDs could be synthesized, as described above. However, the translation of these probes into clinics is somewhat hampered due to their cytotoxic nature (Xiao et al., 2011). Thus, researchers are working to find ways to lower their cytotoxicity, such as using a conventional PDT agent (e.g. porphyrin derivative) to modify the semiconductor QDs and then coat it with a shell consisting of peptides (Tsay et al., 2007). In future, if the use of PDT become feasible for AD treatment then fullerene-based photosensitizers, nanoparticle delivery (Xiao et al., 2011), titania photocatalysis, and upconverting nanoparticles, to enhance the penetration of light into the tissues, could also be employed along with Trojan horse technology to overcome the limitations of existing photosensitizers (Park et al., 2011). In this regard, TfR-targeted gold (Johnsen et al., 2018), and inorganic nanoparticles (Cabezón et al., 2015) have already been shown to accumulate in brain capillaries and further transported across the BBB to enter the brain parenchyma.

## Conclusion

In conclusion, the engineering of a superior molecular Trojan probe is predestined, and scientists will undoubtedly continue working on it. TfRMAb-conjugated molecules, targeted across the BBB should greatly increase the penetration of detection probes or multi-functional neurovascular medicines in cerebral parenchyma to the sites of plaque deposition/development with a resulting greater reduction in the load of cerebral amyloid plaque while leaving normal cells unharmed. This should ultimately result in early diagnosis as well as the improvement in CBF (Yang et al., 2014) and hippocampal-dependent behavioral deficits when tested in transgenic rodent models of AD or AD patients. Trojan technology provides a safe route for the functionalization of photoactive, fluorescent or bioluminescent conjugates to the site of disease epicenter while ensuring the safety of normal tissues. A novel tailor-made Trojan horse system that quickly switches into an aggressive molecule to destroy even residual plaques will be more effective than any known drug thus far developed for AD. Here, we also attempted to propose a novel strategy for inducing photochemical activation and abolish the need for an external light source to activate the photosensitizers or control the ^1^O_2_ production internally; the biggest limitation of light-activated therapies including optogenetics. We also briefly highlighted the feasibility of photosensitizer use for future AD treatments, and to overcome the limitations of existing photosensitizer nanoparticle delivery and targeting. Last, the novel concepts presented here could be potentially beneficial to provide an advanced therapeutic platform for the treatment or diagnosis of other amyloidogenic neurodegenerative conditions, such as Parkinson's disease, Huntington's disease, lysosomal storage disease, and stroke, as well as type-II diabetes, epilepsy, and cancer, with minimal invasiveness.
